# Non-Cell Autonomous Effects of the Senescence-Associated Secretory Phenotype in Cancer Therapy

**DOI:** 10.3389/fonc.2018.00164

**Published:** 2018-05-18

**Authors:** Tareq Saleh, Liliya Tyutynuk-Massey, Emmanuel K. Cudjoe, Michael O. Idowu, Joseph W. Landry, David A. Gewirtz

**Affiliations:** ^1^Department of Pharmacology and Toxicology, Virginia Commonwealth University, Richmond, VA, United States; ^2^Massey Cancer Center, Virginia Commonwealth University, Richmond, VA, United States; ^3^Department of Pharmacotherapy and Outcomes Science, Virginia Commonwealth University, Richmond, VA, United States; ^4^Department of Pathology, Virginia Commonwealth University, Richmond, VA, United States; ^5^Department of Human and Molecular Genetics, Virginia Commonwealth University, Richmond, VA, United States

**Keywords:** senescence, senescence-associated secretory phenotype, chemotherapy, senolysis, dormancy, recurrence

## Abstract

In addition to promoting various forms of cell death, most conventional anti-tumor therapies also promote senescence. There is now extensive evidence that therapy-induced senescence (TIS) might be transient, raising the concern that TIS could represent an undesirable outcome of therapy by providing a mechanism for tumor dormancy and eventual disease recurrence. The senescence-associated secretory phenotype (SASP) is a hallmark of TIS and may contribute to aberrant effects of cancer therapy. Here, we propose that the SASP may also serve as a major driver of escape from senescence and the re-emergence of proliferating tumor cells, wherein factors secreted from the senescent cells contribute to the restoration of tumor growth in a non-cell autonomous fashion. Accordingly, anti-SASP therapies might serve to mitigate the deleterious outcomes of TIS. In addition to providing an overview of the putative actions of the SASP, we discuss recent efforts to identify and eliminate senescent tumor cells.

## Introduction

Therapy-induced senescence (TIS) is a well-established response to conventional cancer therapy (1) that has long been considered a favorable outcome of cancer treatment and a basis for the development of novel therapies that induce a cytostatic response in tumor cells (2). This premise is obligatorily based on what has become a fundamental paradigm relating to senescence, that senescence represents an irreversible form of growth arrest (1). However, we have long postulated that a subpopulation of tumor cells likely can escape TIS and establish tumor re-growth. This is supported by our own work relating to TIS as well as other supporting observations in the literature (3–6). Recent studies by Milanovic et al. strongly suggest that senescence is associated with complex reprogramming that can eventually promote cancer stemness and give rise to a more aggressive phenotype that overcomes the cell cycle blockade (7). In this work, the recovery from senescence could be observed when the maintenance of senescence was no longer active. While multiple mechanisms may prove to contribute to the eventual re-emergence from senescence, this review addresses the potential involvement of the senescence-associated secretory phenotype (SASP).

The SASP is a highly conserved response to genotoxic stress that develops in aging fibroblasts in culture, epithelial cells *in vivo*, as well as tumor cells exposed to DNA-damaging therapeutic agents (8). Senescent cells undergo extensive alterations in gene expression (9); changes that are not only confined solely to genes involved in cell cycle regulation but also include increased expression of a spectrum of secreted proteins (10, 11). Specifically, senescent cells have been reported to secrete a spectrum of pro-inflammatory chemokines and cytokines that have paracrine tumor stimulatory effects (11) contributing to metastatic progression and age-related diseases even in younger cancer survivors (8). However, the non-cell-autonomous effects of the SASP induced by chemotherapy as well as cell-autonomous effects on the tumor microenvironment are not fully understood.

This review focuses on work that supports the contention that the SASP is not only an undesirable outcome of chemotherapy and a major modulator of the tumor microenvironment but also serves as a driver of proliferation that may ultimately be responsible for facilitating escape from senescence. We further suggest that senescence and the SASP may prove to be essential components of tumor dormancy and therefore could serve as critical targets for novel therapies that attenuate the negative impact of senescence on cancer treatment. Accordingly, we also discuss recent approaches to identify senescent tumor cells in patients receiving neoadjuvant chemotherapy and potential approaches for elimination of the senescent tumor cells.

## The SASP is a Response to Cancer Therapy and is Regulated by Cellular Stress Pathways

The SASP can be a direct consequence of the activation of the DNA damage response (DDR) which is uniformly induced in tumor cells treated with chemotherapy or radiation (12, 13) and can also result from oncogene overexpression and hyper DNA replication (14). Interference with DDR-associated proteins such as ataxia–telangiectasia mutant or checkpoint kinase 2 attenuates the senescent response to chemotherapy suggesting that these signaling pathways are critical for TIS (12, 15). p53 is a fundamental regulator of senescence (16), and while it is not an absolute requirement for cells to undergo senescence, loss of p53 function in senescent cells might actually facilitate recovery from senescence (4, 5, 7). This is particularly important given the fact that many tumor cells have mutations that affect the function of p53 (17). Conversely, loss of p53 function can result in enhanced SASP and its ability to drive pro-tumorigenic proliferation (18). The role of p53 in SASP regulation is further shown in senescent hepatic stellate cells implicated in hepatic cirrhosis where p53 ablation attenuates the inflammatory drive mediated by SASP (19). However, the SASP can be precipitated independently of DDR such as in the case of senescent cells that develop non-pathologically during embryogenesis (20) or during wound healing (21). Interestingly, SASP induced by wound healing after mechanical injury lacks IL-6, bFGF, and TGF-β expression (21), whereas several of these factors are expressed after exposure of the lung to DDR-inducing chemicals (22), or from DDR-inducing CCN1 exposure to fibroblasts (23). These observations demonstrate that the composition of the SASP can vary considerably depending on how the senescence is induced.

In addition to DDR and its signaling pathways, cell cycle regulators play important roles in regulating senescence and the SASP. p16^INK4a^ is a tumor suppressor protein that is closely associated with senescence (24). Despite the fact that many tumor cells lose p16^INK4a^ function during transformation, they retain the ability to develop the SASP upon TIS, most likely because SASP is regulated independently of cell cycle arrest (25, 26). This dissociation between SASP and cell cycle regulators is also true for another senescence-associated cyclin-dependent kinase inhibitor, p21^Cip1/Waf1^ (25), although in certain scenarios where SASP is induced independently of DNA damage, p21^Cip1/Waf1^ knockdown can attenuate the secretory response (20).

The SASP is further regulated by p38MAPK which, in addition to p53 and p16^INK4a^ activation, is responsible for an increase in the activity of NF-κB (27). p38MAPK is a primary responder to cellular stress and is activated in response to a variety of anti-tumor agents (28), while NF-κB is responsible for the transcriptional activation of many SASP components and is a chief regulator of TIS (29). In addition to NF-κB, the C/EBPβ transcription factor is necessary for the development of oncogene-induced senescence (OIS) in primary fibroblasts (30). The interplay between C/EBPβ and its heterodimeric partner C/EBPγ regulates the expression of multiple SASP genes (31). Finally, mammalian target of rapamycin (mTOR) appears to play a critical role in the regulation of the SASP as rapamycin promotes a robust suppression of inflammatory mediator release (32). In fact, mTOR inhibition selectively inhibits the translation of the membrane-bound cytokine IL1α, resulting in decreased NF-κB-driven expression of multiple SASP factors (33).

While senescence and SASP are consistently observed in the laboratory in cancer cells both *in vitro* an *in vivo* as a response to DNA-damaging agents (34–36), certain targeted therapies can also induce senescence in tumor cells. For example, the anti-VEGF drug bevacizumab (or inhibition of the VEGFR2 pathway) was able to induce a modest senescent response in colon cancer cells, xenografts, as well as patients’ tumors in a p16^INK4a^ dependent manner (37, 38). In this study, senescence was evaluated based on SA-β-galactosidase staining and p16^INK4a^ expression; however, the ability of bevacizumab to induce SASP expression was not investigated. The effects of anti-VEGF agents on senescent tumor cells are interesting, since VEGF is a core element of the SASP. In fact, bevacizumab in combination with chemotherapy was associated with improved clinical outcomes in glioblastoma patients (39). However, it is not known whether this effect was attributed to enhanced senescence or due to blockade of VEGF as an SASP component.

Recently, aurora kinase inhibitors were shown to induce a robust senescent response in chronic myeloid leukemia, melanoma, and non-small cell lung cancer cells (40, 41). Moreover, CDK4/6 inhibitors such as palbociclib have also been shown to induce a pronounced senescence response in triple-negative breast cancer cells (42). While it is not certain if palbociclib can drive a secretory response in these senescent tumor cells, it was shown that chronic palbociclib treatment promotes senescence and a robust SASP in melanoma-associated fibroblasts which results in enhanced growth of multiple melanoma cell lines (43). This observation is particularly important, since CDK4/6 inhibition is not traditionally associated with DNA damage *per se*, suggesting a possibility for inducing SASP by alternative pathways [in this context, palbociclib has been shown to interfere with DNA damage repair only when tumor cells are exposed to radiotherapy (44)].

Finally, multiple SASP factors do appear to have the potential to fortify the senescent phenotype in a self-reinforcing autocrine fashion. For example, the chemokines receptor CXCR2 (IL8RB) closes the circuit of senescence induced by DNA damage as it conveys NF-κB activation signals by multiple CXCR2-binding factors (45). Furthermore, as a component of the increased expression and release of multiple inflammatory mediators, senescent cells with activated mTOR can actually also show enhanced expression of cytokine receptors such as soluble IL-6R, supporting the premise that the tumor cells could be amenable to self-stimulation (46). Plasminogen activator inhibitor-1 is also a pivotal SASP component that is necessary for the induction and maintenance of the senescent phenotype in fibroblasts (47).

**Figure 1:**
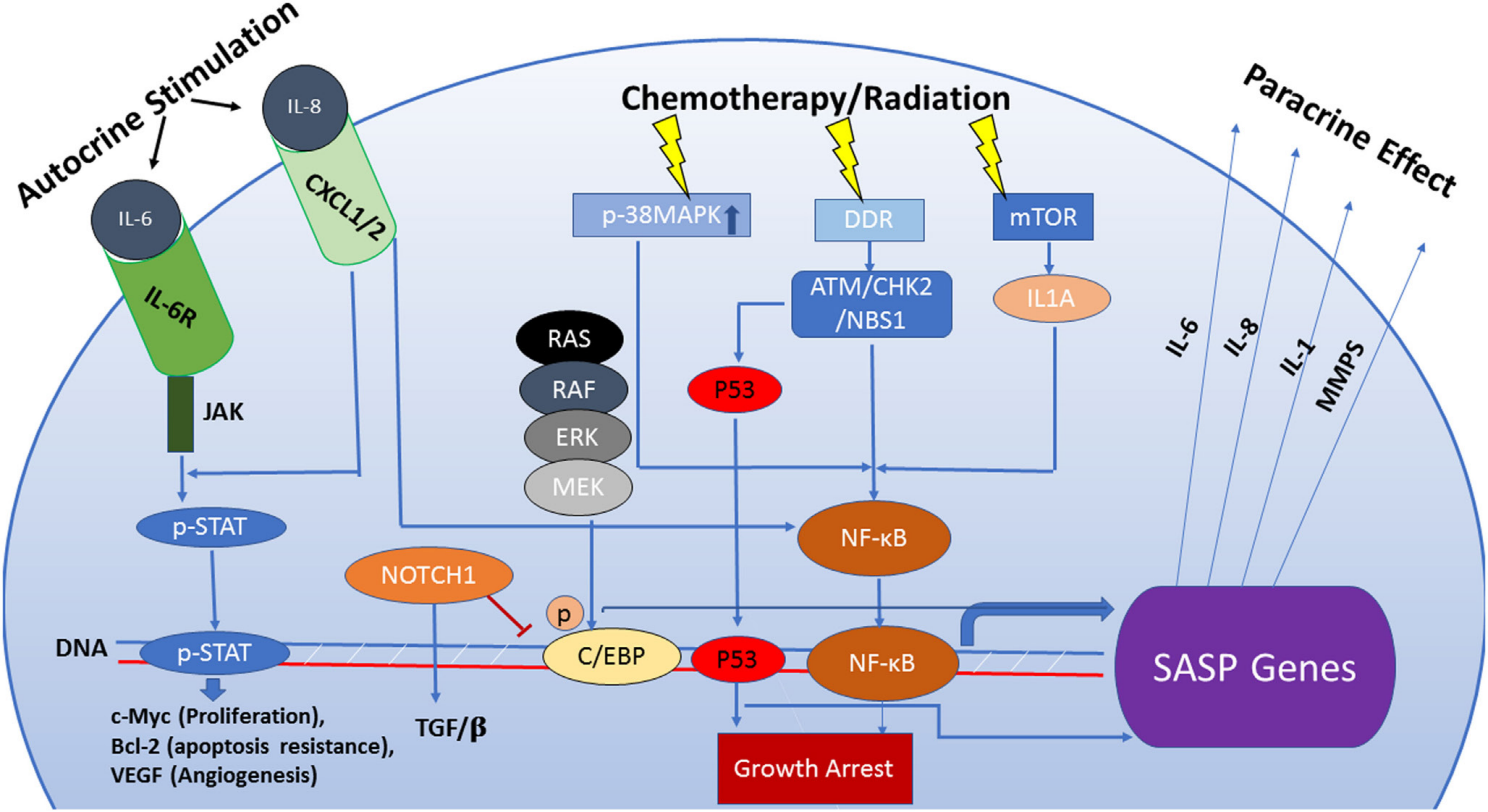
The senescence-associated secretory phenotype (SASP) is regulated by an interactive network of pathways that dictate its functional outcome. Persistent DNA damage can be responsible for activation of the SASP by a mechanism that is independent of cell cycle regulation. Also, the SASP can be induced via pathways separate from the DNA damage response (DDR) such as activation of p38MAPK or mTOR which, in turn, enhance transcriptional activity of NF-кB. Increased transcription of SASP genes is predominantly regulated by the transcription factors NF-кB and C/EBPβ. The composition of the SASP is regulated by genotoxic stress pathways, mainly the p53 tumor suppressor pathway. p53 attenuates the SASP through regulation of NF-кB function but can also directly influence the transcription of SASP genes qualitatively, facilitating immune clearance of senescent cells. NOTCH1 signaling modulates the secretome of senescent cells by inhibiting C/EBPβ, thus suppressing pro-inflammatory secretion in favor of a TGF-β-rich composition. This switch prevents recruitment of the immune cells to the tumor site. Components of the SASP act as signaling molecules in both paracrine and autocrine fashion and are capable of stimulating complex and sometimes conflicting pathways including senescence maintenance or escape, immune system activation or suppression, induction of tumorigenesis or tumor suppression in neighboring cells.

Despite the extensive body of literature relating to the SASP, it is uncertain whether the SASP contributes to the maintenance of senescence or promotes escape from senescence (Figure 1). In fact, the dynamic interplay of the different regulatory pathways of SASP can yield different functional outcomes. For example, the oscillation of NOTCH1 expression and signaling during transition into OIS can culminate into two distinct SASP profiles with variable effects (either an anti-inflammatory TGF-β dominate or a pro-inflammatory SASP response) (48). To summarize, the outcome of the SASP is dependent on the heterogeneity of the senescent response (49), the profile of secreted factors dictated by the interplay of the SASP’s regulatory pathways, cell type, temporal status of senescence, and interaction with other components of the microenvironment (50).

## Non-Cell Autonomous Contributions of the SASP to Senescence, Malignancy, and Response to Therapy

### The Effect of SASP Derived From Senescent Fibroblasts and Senescent Stromal Cells

It is well established that the SASP in normal cells such as fibroblasts can promote tumor growth in a paracrine or non-cell-autonomous fashion (51, 52). For example, senescent fibroblasts are capable of enhancing tumor xenograft growth in animals (53) and accelerating pre-neoplastic cellular growth by both direct cellular contact as well as the release of soluble factors in the vicinity of the tumor cells (54). Regardless of how senescence is induced (by oncogene overexpression, oxidative stress, or replicative exhaustion), these fibroblasts are capable of promoting this tumorigenic effect (54). This suggests that chemotherapy and radiation might also stimulate a pro-tumorigenic response in the tumor microenvironment *via* senescence induction. In fact, a short exposure to chemotherapy can induce senescence in cancer-associated fibroblasts (CAFs) accompanied by a robust inflammatory phenotype (55). These senescent CAFs can promote enhanced tumor cell growth, invasion, migration, and possibly distant dissemination (55, 56).

Multiple elements of the SASP are implicated in the induction of the epithelial–mesenchymal transition (EMT), which contributes to enhanced invasiveness of the developing epithelial tumor (57). Moreover, senescent fibroblasts promote angiogenesis, which is essential for tumor growth and sustainability (58). In addition, the SASP is strongly implicated in the induction of a cancer stem cell-like phenotype following tumor cell exposure to DNA damage (59). This paracrine effect mediated by tumor stromal cells or aging fibroblasts is definitely deleterious and would not only influence tumor behavior but also the response to cancer therapy and overall treatment outcome. Accordingly, since the SASP can act in a paracrine fashion to drive the proliferative phenotype, it is reasonable to postulate that the SASP also has the capacity to act in an autocrine (cell-autonomous) fashion to confer proliferative capacity upon the senescent cells.

On the other hand, it has been suggested that senescent fibroblasts favor the accumulation of more senescent cells in the neighboring tissue (60). This bystander effect was attributed to the ability of these cells to induce the activation of the DDR in non-senescent fibroblasts (60). Here, instead of secreting soluble factors, senescent fibroblasts were able to induce senescence *via* gap junction-mediated intercellular contact (60). The major driver of this bystander effect was strongly connected to mitochondrial dysfunction and ROS generation, which not only stabilizes the senescent state but also induces senescence in a neighboring cell (61). Furthermore, NF-κB blockade was sufficient to abrogate this bystander effect, again, highlighting its pivotal regulatory role in senescence (61).

### The Effect of Tumor Cell Derived SASP in Response to Cancer Therapies

As discussed thus far, the effects of senescent fibroblasts on tumorigenesis and tumor progression have been investigated quite extensively, establishing the pro-tumorigenic role of the SASP in the tumor microenvironment, where it favors increased aggressiveness of a growing tumor. However, the role of the SASP induced in tumor cells when exposed to chemotherapy or radiation is not often addressed and its autocrine and paracrine impact on tumor cells or stromal fibroblasts in juxtaposition is not fully elucidated. We have reported previously that conditioned medium from breast tumor cells exposed to adriamycin can induce a senescent growth arrest in naïve breast tumor cells, suggesting, at least initially, that SASP maintains the senescent phenotype in both autocrine and paracrine fashion (62). Subsequently, this bystander effect was shown to be mediated by insulin-like growth factor binding protein 3 (63). By contrast, senescent prostate cancer cells promoted the proliferation of bystander tumor cells, consistent with the more well-established role of the SASP (64). However, this effect was less robust than the ability of senescent fibroblasts to affect neighboring cells in a paracrine fashion. In addition, the tumor stimulatory effect was lost *in vivo*. Conversely, the adriamycin-induced bystander effect resulted in decreased chemosensitivity (but not radiosensitivity) in HeLa cells (65), indicating that SASP might have a more complex effect on tumor growth and responsiveness to therapy. For example, it is reported that SASP inhibition by NF-κB inactivation is associated with chemoresistance and relatively poor survival (29). By contrast, the SASP of cancer cells exposed to chemotherapy has been shown to generate a chemoresistant, more invasive cell population which attenuates the responsiveness of cancer cells to treatment (66). The ability of the SASP to confer resistance to apoptosis can be exploited therapeutically using senolytic agents and has become an area of intense research (discussed below). We should also re-emphasize that senescent tumor cells themselves are resistant to apoptosis by definition and can evade the potential cytotoxicity of chemotherapy and radiation (67).

Clinically, cancer patients developing senescent tumors following neoadjuvant chemotherapy showed a significant decrease in survival (68). Furthermore, meta-analysis data collected over the years consistently shows a correlation between elevated levels of SASP pro-inflammatory cytokines and poor prognosis in patients with different types of cancer. For example, in colorectal cancer, IL-6 is associated with a high probability of tissue invasion and metastasis, and is viewed as a predictor of low overall survival (69, 70). Similarly, in breast, prostate, lung, and ovarian cancers, elevated levels of IL-6 and IL-8 have been found to correlate with progression to advanced stage and development of metastatic disease (69, 70). Considering the apparent importance of these two cytokines to survival and prognosis and their relevance in TIS, we provide a more detailed discussion of their regulation and contribution to the senescence response to cancer therapy.

### IL-6 and IL-8 Are Pivotal Components of the SASP Response to Cancer Therapy

The SASP resulting from exposure to cancer therapy shares common secreted mediators with the other forms of senescence, most prominently the two soluble chemokines IL-6 and IL-8 (71). Both of these cytokines, among other SASP components, have been reported to be expressed at higher levels from tumor cells of patients who received chemotherapy (e.g., prostate cancer patients treated with mitoxantrone) in association with increased p16^INK4a^ and p21^Cip1/Waf1^ levels, indicating that senescence and its secretory phase are clinically relevant responses to chemotherapy (18, 68).

Il-6 and IL-8 are essential mediators of inflammation and wound healing and are responsible for increasing the blood supply, mobilizing leukocytes to the sites of tissue damage, and promoting tissue repair (72, 73). Cancer-associated inflammation facilitates proliferation of oncogene-transformed cells and their spread to distant sites *via* lymphatic or vascular systems (74). In this case, IL-6 and IL-8 signaling can lead to tumor initiation, progression, and metastasis.

IL-6 is a pleiotropic cytokine produced by multiple cell types that is notably upregulated in senescent cells. IL-6 functions through the activation of JAK tyrosine kinase which results in the phosphorylation of the signal transducer and activator of transcription (STAT3) (75). STAT3 activates anti-apoptotic proteins of the Bcl2 family, the transcription factor c-Myc (responsible for cell cycle reentry and cell proliferation), VEGF (responsible for angiogenesis), and vimentin and fascin (involved in metastatic progression) (76, 77). The STAT3 pathway is involved in cell survival, cell proliferation, as well as progression through the cell cycle (77). Most importantly, STAT3 promotes upregulation of both the IL-6 and IL-8 receptors, indicative of a potential autocrine, cell-autonomous regulation of senescence (78).

Early on during tumorigenesis, IL-6 is produced primarily by the stromal cells and activates the JAK/STAT3 pathway in neighboring tissue (79, 80). Cells that have undergone malignant transformation stimulated by IL-6 signaling subsequently increase secretion of the cytokine IL-6, further activating the growth promoting JAK/STAT axis (81). As mentioned earlier, the tumorigenic actions of IL-6 are not only limited to paracrine stimulation but also include malignant transformation through autocrine signaling (82).

IL-6 is known to activate the NOTCH signaling pathway in an autocrine fashion (83). In addition to being involved in senescence regulation, NOTCH is responsible for acquisition of stem cell features, epithelial to mesenchymal transition (anchorage-independent growth, resistance to anoikis) and the ability to survive under hypoxic conditions (84).

IL-8 is a ligand for two G-protein coupled receptors (CXCL1 and CXCL2) (85). CXCL1/2 signaling results in activation of a vast network of downstream effectors such as protein kinases responsible for cell survival and proliferation, transcription factors such as NF-κB that mediate protein expression, and GTPases that orchestrate cytoskeleton remodeling (85). The IL-8-induced signaling cascade leads to enhanced translation of cyclin D1, a protein responsible for cell cycle progression from G1 to S phase (85). IL-8 stimulation of its receptor also results in phosphorylation and activation of JAK2 and STAT3 independently of the IL-6 receptor reinforcing IL-6 signaling (85). Both IL-6 and IL-8 can act in concert to induce growth of primary tumors and promote anchorage-independent growth essential for metastatic progression (86–88).

Activation of VEGFR is necessary for the induction of vascular permeability (89). During inflammation, IL-8 serves as a chemotactic factor attracting neutrophils to the site of tissue damage where increasing vascular permeability is necessary (90). However, during cancer progression, leaky vessels allow extravasation of cancer cells that contributes to metastatic progression (91, 92). Moreover, IL-8 is an established factor that drives epithelial to mesenchymal transition in cancer cells (93). In the literature, there is a strong correlation between high expression of IL-8 and mesenchymal and stem cell features in tumor cells known for their aggressive behavior (94, 95). Furthermore, treatment with conditioned media from cells secreting high levels of IL-8 induces epithelial-mesenchymal transition (EMT) in naive cells, while on the other hand, application of an IL-8 inhibitor leads to loss of mesenchymal markers and development of an epithelial phenotype (93).

## TIS, SASP, and the Immune System

It is now appreciated that selected cytotoxic chemotherapies and radiation can enhance tumor cell immunogenicity by promoting immunogenic cell death (ICD) (96). A requirement of ICD is therapy-induced autophagy which, when left unresolved, results in apoptosis. This process of autophagic cell death promotes the secretion of damage-associated molecular patterns which stimulate antigen-presenting cells to cross present antigens liberated from the cell death (97). However, under conditions where TIS results in an immune suppressive SASP, the benefits of ICD can be lost. This could happen frequently as therapy-treated tumors will likely contain a mixture of cells undergoing immune stimulating ICD and immune suppressive TIS (98).

Generally, in those circumstances where therapy induces ICD, the immune response can eliminate the tumor cells (99). However, TIS is less deterministic as it can promote both pro- and anti-tumor immune activities (100). Central to the variability of the immune response to TIS is the SASP (8). The SASP is capable of attracting and further differentiating a variety of pro- or anti-tumor immune cells in the tumor microenvironment including natural killer (NK) cells, neutrophils, monocytes/macrophages, and T cells (101). How immune cells respond to the SASP depends on which chemokines are secreted, which, in turn, depends on how the tumor cell responds to chemotherapy (102). Depending on this response, SASP can lead to the elimination or the protection of tumors cells, where the latter could represent a key element for maintaining tumor cell dormancy and its potential recurrence (103). However, the consequences of TIS to the anti-tumor immune response are less well characterized, largely due to difficulties in generating a pure population of senescent cells to study, in contrast to OIS, which has been studied in multiple contexts and has been reviewed extensively (104).

Just how variable the SASP response can be was recently demonstrated for MYCN neuroblastomas (36). In this study, 12 chemotherapies targeting a wide range of cellular targets were screened for their ability to induce TIS of MYCN amplified neuroblastomas followed by analysis of the SASP output from a subset of these therapies. From this screen, low-dose topotecan was determined to induce senescence and a cytokine profile predicted to stimulate an anti-tumor response. By contrast, BrdU-induced senescent cells induced a pro-tumorigenic SASP response which is ultimately permissive to tumor growth. As predicted from this screen, treating MYCN amplified tumor in mouse models with topotecan resulted in TIS, a favorable SASP, and complete or partial tumor remission; however, its effects on immune cell recruitment was not analyzed.

In most cases TIS results in an SASP that promotes an anti-tumor response, leading to tumor cell elimination. In human melanoma xenografts, inhibition of aurora kinases using MLN8237 resulted in impaired mitosis, DNA damage, and induced an IKKb/NF-κB-dependent TIS and the accompanying SASP (105). The SASP resulted in increased neutrophil and macrophage recruitment into the tumor, which promoted clearing the therapy treated tumor cells. To introduce rigor, a mouse MelA melanoma tumor model in an immune competent model was used to demonstrate that tumor growth control required macrophages. Dendritic cells and macrophages are commonly found in senescent tumors and originate from circulating monocytes which are recruited to senescent tumors by a variety of cytokines (106). Once localized, exposure to SASP cytokines can differentiate monocytes into macrophages and dendritic cells. SASP factors such as IFNγ and IL-6 skew macrophage polarization toward the anti-tumor M1 macrophages. Likewise IL-4 and IL-13 cytokines of the SASP response polarize macrophages to the tumor-promoting M2 macrophages (107). In a follow-up study, the authors showed that alisertib (another aurora kinase inhibitor) results in immune cell recruitment by secreting the chemokine CCL5 (98). The immune response to alisertib was further enhanced with a CD137 agonist, but not significantly to untreated tumors, to promote survival of antigen primed T cells, suggesting that TIS and the SASP improved tumor immunogenicity.

In addition to myeloid immune cells, NK and T effector cells do react with cells undergoing TIS. In a combination therapy strategy, radiation-treated tumors were also treated with the PARP inhibitor veliparib to inhibit DNA damage repair, thereby enhancing TIS (108). Melanoma and pancreatic tumors treated with this combination therapy express an immune stimulatory SASP, most significantly CCL5, IFN-β, and CXCL11, to promote dendritic cell proliferation and enhanced cytotoxic lymphocyte anti-tumor activity. Depletion studies identified CD8 T cells and NK cells as important effector cells for the anti-tumor response to TIS from this drug combination (98). These T cell responses could also result from major histocompatibility-dependent mechanisms which have been observed in models of OIS (109).

Natural killer cells play a central role in the recognition of senescent tumor cells. In this capacity, the NK cells recognize a number of SASP inflammatory cytokines through their diverse array of cell surface receptors (110, 111). Once NK cells detect these cytokines, they rapidly infiltrate into the senescent tumor. Common to the SASP response is IL-15, an NK cell activating cytokine which promotes the anti-tumor functions for NK cells (111). Because of their ability to be recruited to tumors and be activated, NK cells have prominent roles in the immune response to TIS irrespective to the variability in the SASP response of different tumors cells to therapy (112). Once recruited, NK cells recognize NKG2D and DNAM1 stress ligands upregulated in response to therapy-induced DNA damage and associated TIS (113, 114). In addition to stress ligands, TIS cells upregulate adhesion molecules including ICAM-1 which interacts with the CD58 receptor on NK cells to promote tumor cell–NK cell contact—a prerequisite to tumor cell killing (115) NK cell-mediated toxicity of senescent cells is primarily through the granule exocytosis pathway and not Fas/FASL (116). Like NK cells, T cells can be mobilized, activated, and differentiated in the senescent tumor from a variety of SASP factors including CCL27, CCL2, CCL5, CXCL11, and IL-1α (117). Variability in other cytokines secreted as part of the SASP response can recruit either Th1 or Th2 CD4 T cells and maintain their polarization in the tumor. The consequences of these functions can promote and maintain either a pro-tumor Th2 or anti-tumor Th1 microenvironment (118). In addition to promoting CD8 T cell recruitment, therapy-induced DNA damage and associated upregulation of NKG2D ligands can also stimulate the cytotoxic effects of CD8 T cells through the NKG2D receptor (119).

By contrast, a pro-tumorigenic SASP response from the myeloid immune compartment was demonstrated in prostate cancers. The implications for a pro-tumorigenic SASP response to TIS are more straightforward. In one example, PTEN-deficient prostate cancers treated with docetaxel undergo TIS and induce a corresponding immune inhibitory SASP response by amplifying myeloid-derived suppressor cells (MDSCs). Amplification of MDSCs renders the tumor refectory to the therapeutic effects of docetaxel. Enhanced MDSC recruitment and activity is a well-known means which tumor cells suppress the anti-tumor immune response to survive in the patient (120). Inhibition of the JAK2/STAT3 axis in these tumors removes the immune inhibitory SASP response to docetaxel, the accompanying MDSCs, and improves therapeutic outcomes (121). Subsequent studies showed that reducing myeloid cell recruitment using a CXCR2 antagonist improved the ability of docetaxel to induce TIS, thereby enhancing its therapeutic effects (122). The immune suppressive nature of TIS in prostate cancer cells is also supported using the TRAMP-C2 prostate cancer cell model. When this model is induced into TIS with docetaxel, the resulting senescent cells suppress the immune response to co-transplanted tumor cells (123). This immune suppression could be blocked by expressing IL-12 in the tumor, suggesting that immunotherapies could break the immune suppression and improve therapeutic outcomes for prostate cancers.

As discussed above, IL-6 is a major component of SASP that functions predominantly *via* the JAK/STAT3 pathway. In addition to its pro tumorigenic activities, phosphorylated STAT3 is involved in recruitment and differentiation of Tregs and MDSCs which suppress CD8^+^ T cells (124). At the same time, STAT3 facilitates inhibition of dendritic cell maturation and deactivation of T cells and macrophages (125, 126). Perhaps of greatest clinical relevance, activation of STAT3 through IL-6 signaling allows cancer cells to escape immune surveillance and can prevent clearance of senescent cells by the immune system (127).

This complex interaction between senescent tumor cells and the immune system makes it challenging to draw direct conclusions with regard to the contribution of TIS to the recovery from tumor dormancy and outgrowth. Under most conditions, when an anti-tumor SASP response occurs, escape from therapy-induced tumor dormancy would require that TIS, and its accompanying anti-tumor SASP response, both be reversible. Conversely, in rarer circumstances, documented mostly for prostate tumors, a pro-tumor SASP response could lead to a suppressed immune response and the maintenance of dormant tumor cells (121–123).

## The Potential for Anti-SASP and Elimination of Senescent Tumor Cells as a Therapeutic Strategy

### Anti-SASP Therapy

Senescence has long been considered an adventitious outcome of cancer treatment based on the fact that chemotherapy and radiation induce an initial, senescent, and cytostatic delay in tumor growth (128). This has basically served as the basis for the utilization and development of pro-senescence therapy (129). However, it is feasible that senescence may actually represent an undesirable outcome of therapy in that senescent tumor cells can potentially remain dormant for extended periods of time, only to resume proliferation at some later date, thereby contributing to, if not playing a central role in, cancer recurrence, disease morbidity, and mortality. In fact, the expression of senescence markers in patients receiving neoadjuvant chemotherapy and/or radiation has been associated with poor prognosis (68, 130–132). Furthermore, it is well established that the SASP contributes to unfavorable outcomes, such as adverse effects of chemotherapy (133). Accordingly, several approaches have been proposed to attenuate the deleterious effects of the SASP (summarized in Table 1A).

**Table 1:** Potential anti-senescence-associated secretory phenotype (SASP) and senolytic therapies.

**(A)**	
**Targeted pathway**	**Modality**

Broad anti-inflammatory	Glucocorticoids, e.g., corticosterone, cortisol (134)
Mammalian target of rapamycin (mTOR)	Rapamycin (32, 33)
Rho family GTPases	Simvastatin (135)
p38MAPK/MK2	UR-13756 and BIRB 796 (136)
IL-6 receptor	Toclilizumab, siltuximab (50)
IL-1 receptor	Anakinra (50), rilonacept
NF-кB	Metformin (137)

**(B)**	
**Targeted pathway**	**Method of elimination**

Bcl2 anti-apoptotic family	ABT-263 (Navitoclax) (138)
	ABT-737 (139)
	A1331852 (140)
	A1155463 (140)
Cell surface glycoproteins, DPP4	Elimination by antibody-dependent cell-mediated cytotoxicity (ADCC) (141)
Heat shock protein 90 (HSP90)	17-DMAG (alvespimycin) (142)
PI3K/Akt survival pathway	Quercetin (143)
	Fisetin (140)
Tyrosine kinase receptors	Dasatinib (143)
Glucose and fatty acid metabolism, AMPK, autophagy	Phloretin (144)
	Cytochalasin B (144)
	Sodium oxamate (144)
	Etomoxir (144)
	Compound C (144)
Histone deacetylase inhibitors (HDACs)	Panobinostat (145)

*A. Summary of drugs that have attenuated the SASP or potential targets for anti-SASP therapy*.

*B. Summary of drugs that demonstrated the ability to clear senescent cells either in vitro or in vivo, indicative of possible targets for the elimination of senescent cells*.

Broad-spectrum anti-inflammatory substances such as glucocorticoids have been shown to effectively suppress certain aspects of the SASP (134). Despite exerting their effects without interfering with the tumor suppressive component of senescence, wide-range anti-inflammatory drugs have variable adverse effects. Accordingly, more specific approaches would be desirable. For example, mitochondrial integrity is essential for the regulation of certain features of the senescent phenotype including its secretory phase, and targeting the mitochondria and proteostatic pathways has been proposed as a potential anti-SASP treatment (144). In fact, targeting autophagy and glucose utilization processes in senescent tumor cells that are engaged in highly active secretory phase is proposed as a novel approach to combat the negative effects of senescence during cancer therapy (144). The mTOR inhibitor, rapamycin, results in direct inhibition of IL-1α synthesis (and consequently IL-6 secretion) which successfully prevented the tumor-promoting effects of senescent fibroblasts (33).

Simvastatin, a common antihyperlipidemic agent, promotes a robust reduction in the activity of CDC42, which, in turn, decreases IL-6 secretion by senescent cells (135). It has thus been suggested that simvastatin might actually suppress breast cancer resistance that develops due to the accumulation of senescent cells following chemoradiation (135). Direct inhibition of p38MAPK has also been associated with a robust inhibition of SASP, providing another pathway for small molecule targeting (136). Finally, several interleukin inhibitors, available not only experimentally but also clinically, can be utilized in combination with chemotherapy. For example, tocilizumab (IL-6 receptor antibody) and anakinra (IL-1 receptor antagonist) are used as immunomodulating therapy in the treatment of inflammatory diseases and can be readily investigated as regulators of the SASP in combination with conventional cancer treatment (50).

However, the challenge will be to therapeutically alter the SASP output to improve, rather than inhibiting tumor cell immunogenicity. Success with this strategy has been achieved in the clinic using TGFβ neutralizing antibodies, a key component of the SASP, in combination with radiation (146). Using a similar strategy, additional immune suppressive cytokines could be targeted. Additional enhancement could occur when combined with immune suppressive checkpoint blocking therapies which have revolutionized the treatment of specific tumors types including melanoma and lung cancer (147). These successes make it plausible that SASP and therapy-induced immunogenic cell states can be exploited for therapeutic benefit.

### Elimination of Senescent Cells by Senolytic Agents

To eliminate these cells, and collaterally suppress the SASP, efforts have been focused on developing senolytic agents; compounds that selectively clear senescent cells without compromising the viability of their healthy neighbors (148) (summarized in Table 1B). This approach is primarily based on promoting apoptosis in senescent cells, which otherwise appear to be capable of resisting cell death and maintaining survival for an extended period of time (149). For example, senescent cells upregulate anti-apoptotic pathways, theoretically making them susceptible to cell death by interfering RNA or small molecules that target these pathways (143). Furthermore, the SASP itself has been postulated to confer apoptosis resistance in senescent cells (150).

Recently, a novel approach to selectively eliminate senescent cells in mice has paved the way for a more efficient pharmacological clearance of such aging cells (138). This is based on the fact that many senescent cells upregulate the expression of Bcl2 family proteins, which serves to attenuate or suppress apoptosis. Bcl2 inhibitors such as ABT-737, ABT-263, or histone deacetylase inhibitors that act to reduce expression of Bcl2 proteins have been shown to effectively target senescent cells (138, 139). Elimination of senescent cells had a positive impact on certain age-related phenomena (151). One could argue that combining these therapies with conventional pro-senescence cancer therapy might actually improve treatment outcome. In fact, the Bcl2 inhibitor, ABT-737, used in combination with docetaxol, has been shown to be effective in sensitizing tumor cells to chemotherapy in mouse primary xenografts (152). In a hepatoblastoma model, ABT-737 administered with either adriamycin, etoposide, or paclitaxel *in vitro* enhanced drug cytotoxicity, which can prevent the development of multi-drug resistance (153). Furthermore, Bcl2 inhibition was associated with positive outcomes both *in vitro* and *in vivo* in studies of acute lymphoblastic leukemia (154). While this approach appears promising, a major challenge lies in the fact that Bcl2 upregulation in senescent cells is not a universal phenomenon and some senescent cells might not be amenable to senolysis. Accordingly, efforts have been directed toward the identification of druggable senescence-specific targets, such as the cell surface protein DPP4 (141) or the heat shock protein 90 (142).

## How Can We Identify Senescent Tumor Cells or Tumor Cells Predisposed to Senescence Clinically?

From a clinical and pathological standpoint, the pathogenesis and significance of senescence is important because of its potential relationship to cancer recurrence. As discussed earlier in this review, cancer patients are often treated with radiotherapy and chemotherapy, which may be used in the neoadjuvant or adjuvant settings, and these treatments may induce tumor senescence. Because the SASP response can vary in composition, developing means to detect its presence in patients as a diagnostic has not been pursued. For this approach to be successful universal markers of the SASP must be identified. Initial studies have discovered an SASP-responsive alkaline phosphatase which, if subsequent studies show is commonly found in tumor cells, could fulfill this requirement (155). However, in the absence of a universal SASP marker, more traditional senescence markers must be used. Both p53 and p16^INK4a^ have been implicated in OIS as a protective mechanism (156, 157). Although mutated in most tumors, it is believed that some subset of carcinomas may still be able to use p16^INK4a^ and p53 associated pathways to induce senescence (158). Here, we describe several approaches to identify senescent tumor cells in the clinic.

Senescent tumor cells cannot be distinguished from non-senescent tumor cells histologically using the routine hematoxylin and eosin (H&E) stain. Ancillary stains such as immunohistochemistry are often necessary to identify senescent tumor cells. Given that senescent tumor cells arrest in the G1 or G2/M phase of the cell cycle, it stands to reason that there may be variable expression of some of the cell cycle protein regulators (in G1 phase, G1/S checkpoint, G2 phase, and GS/M checkpoint) such as p16^INK4a^, p21^Cip1/Waf1^, and p27 to achieve and maintain growth arrest. Antibodies targeting these regulator proteins may be useful in detecting senescent tumor cells or cells predisposed to tumor senescence (159). Ideally, evaluating the pre-treatment and post-treatment tumor samples should show an increase in residual senescent tumor cells post neoadjuvant therapy. It has, however, been reported that tumor predisposed to senescence may be discovered prior to treatment and that this might in fact affect treatment outcome (160).

Senescence-associated β-galactosidase (SA-β-gal) has been shown to be an excellent marker for the evaluation of senescence (161, 162). However, the need for cell culture and fresh or frozen tissue limits the utility of SA-β-gal for routine clinical use as a marker of senescence, since most of the clinical specimens are formalin fixed paraffin embedded (FFPE) tissues. A fully annotated tissue bank is an excellent resource for frozen tissue; however, tissues for banking are usually residual tissue after the specimens have been sampled for pathologic diagnosis and staging. In some cases, especially in the setting of neoadjuvant treatment, there may not be residual tissue for banking. To eliminate the limitation of using SA-β-gal in FFPE samples, immunohistochemistry expression of lysosomal β galactosidase (GLB1) in FFPE samples has been shown to correlate with the senescent phenotype (163).

Histochemical markers to detect lipofuscin in tumor have also been suggested as markers of senescence (164). Lipofuscin is an aggregate of oxidized proteins and lipids which accumulate as part of the physiologic aging process (165) and its accumulation may be seen in different organs. Lipofuscin may be readily seen using routine H&E stains in the liver and other organs, where it may mimic iron pigments; negative Prussian blue histochemical for iron usually differentiates between lipofuscin and iron. In cases where it is not readily apparent, the following histochemical stains have been suggested as lipofuscin markers: Fontana-Masson, eosin, Sudan Black, Berlin Blue, Ferric Ferrycyanide, and prolonged Ziehl–Neelsen (165–167). Sudan Black is usually used to identify adipose tissue, while Ziehl–Neelsen is usually used for the detection of acid-fast organisms. From our limited experience, we have found these stains to be adequate in highlighting lipofuscin. However, it must be pointed out that prolonged Ziehl–Neelsen staining (longer time in carbol fuschin) is better for lipofuscin. The use of markers that are already commonly used in most clinical laboratories for evaluation of patients’ samples will facilitate their adoption for use in tumor senescence.

The utility of lipofuscin detection is questionable in actively dividing tumor cells given that lipofuscin accumulation is readily seen in post-mitotic (non-actively) dividing cells such as liver, skeletal muscle, neurons, and retinal epithelium and very much diluted (not readily seen) in actively dividing cells of intestinal epithelium and bone marrow (168, 169). However, given that senescent tumor cells are not actively dividing, it may be possible to detect lipofuscin, though the sensitivity is unclear. Considering these limitations, immunohistochemical stains (p53, p16^INK4a^, p21^Cip1/Waf1^, and p27) may currently be preferable in the evaluation of senescence.

## Conclusion

Overall, senescence and its secretory phenotype are both pivotal responses of tumor cells to cancer therapy. While the role of the SASP in the pathogenesis of degenerative disease and tumor development is well established, the manner whereby the SASP influences tumor senescent cells, their response to further therapy and their ability to escape the senescent state remains to be fully elucidated. Many of the SASP factors are strong pleotropic mediators that drive cellular proliferation and can possibly facilitate reentry into the cell cycle of senescent cells in a non-cell autonomous fashion. However, the effect of the SASP on premalignant tissues is likely to be different from how the SASP would influence tumor cells that are in an established fully senescent response. Also, in the tumor microenvironment, the interplay between the SASP of stromal cells and the SASP of therapy-exposed tumor cells is likely to be more complex, particularly as the SASP is also involved in tumor immunogenicity. Nevertheless, in the context of developing a better understanding of SASP’s role in tumor progression, we hypothesize that many SASP mediators, such as IL-6 or TGFβ, can be drivers of cancer recurrence both by inducing the re-growth of tumor senescent cells and facilitating their escape from immunosurveillance.

Escape from senescence mediated, in part by the SASP, is likely to have substantial clinical implications. After successful surgical resection, remnant senescent tumor cells can persist for extended periods of time. These cells engage in active secretion of multiple SASP factors that can facilitate the dormant state (e.g., promoting angiogenesis and ECM modulation). Eventually, some senescent tumor cells may manage to escape the growth arrest and re-emerge as actively proliferating cells that contribute to cancer recurrence. As a therapeutic strategy, we propose the routine assessment of senescence induction following neoadjuvant therapy using the methods described above. This allows for outcome assessment as well as the administration of novel therapies that can eradicate the remaining senescent cells. If senescent cells were successfully eliminated, their contribution to the adverse effects of chemotherapy through SASP would be diminished, and more importantly, their role in facilitating tumor dormancy could be significantly lowered. Until these modalities have been fully characterized, available anti-SASP therapy can potentially be combined with conventional cancer treatment to mitigate the deleterious effects of the multitude of factors secreted by the senescent tumor cells.

## Author Contributions

TS contributed to the conceptual proposal, conclusions, writing and editing of the main sections, and did the majority of literature review. LT-M contributed to writing and editing of the section on the SASP in tumor cells and roles of specific SASP factors. EC contributed to the writing, editing, and literature review. MI provided clinical guidance and contributed to the writing of the section on clinical evaluation of senescent tumor cells, development of novel methodology to identify them in patients receiving neoadjuvant chemotherapy. JL provided guidance and writing on the role of the immune system in clearance of senescent tumor cells and ways to enhance their immunogenecity. He also described the role of the SASP in affecting immunosurveillance. DG provided guidance, conceptual supervision, as well as contributed heavily to the writing, editing, and shaping the manuscript in its current form.

## Conflict of Interest Statement

The authors declare that the research was conducted in the absence of any commercial or financial relationships that could be construed as a potential conflict of interest.
